# Growth of Global Publishing Output of Health Economics in the Twenty-First Century: A Bibliographic Insight

**DOI:** 10.3389/fpubh.2017.00211

**Published:** 2017-08-11

**Authors:** Mihajlo (Michael) Jakovljevic, Ana V. Pejcic

**Affiliations:** ^1^Health Economics and Pharmacoeconomics, Faculty of Medical Sciences, University of Kragujevac, Kragujevac, Serbia; ^2^Faculty of Medical Sciences, University of Kragujevac, Kragujevac, Serbia

**Keywords:** health, economics, interdisciplinary, bibliography, literature, INTREPID

## Abstract

**Background:**

Strong growth of interdisciplinary sciences might find exceptional example in academic health economics. We decided to observe the quantitative output in this science since the beginning of the twenty-first century.

**Methods:**

Electronic search of the published literature was conducted in four different databases: one medical database—MEDLINE/PubMed, two general databases—Scopus/Elsevier and Web of Science (WoS), and one specialized health economic database—NHS Economic Evaluation Database (EED). The applied combination of key words was carefully chosen to cover the most commonly used terms in titles of publications dealing with conceptual areas of health economics. All bibliographic units were taken into account.

**Results:**

Within the time horizon from January 1, 2000 to December 31, 2016, without language or limitations on bibliographic unit types, we identified an output ranging approximately from 60,345 to 88,246 records with applied search strategy in MEDLINE/PubMed, Scopus/Elsevier, and WoS. In NHS EED, we detected 14,761 records of economic evaluations of health interventions during the period in which database was maintained and regularly updated. With slightly more than one-third of the identified records, USA clearly dominates in this field. United Kingdom takes a strong second place with about 12% of identified records. Consistently, USA and UK universities are the most frequent among the top 15 affiliations/organizations of the authors of the identified records. Authors from Harvard University contributed to the largest number of the identified records.

**Conclusion:**

There is a clear evidence of both the upward stream of blossoming in health economics publications and its acceleration. Based on this bibliographic data set, it is difficult to distinguish the actual impact growth of this output provided dominantly by academia with modest contribution by pharmaceutical/medicinal device industry and diverse national government-based agencies. Further insight into the citation track record of these individual publications could provide helpful upgrade and a perspective on ongoing development.

## Introduction

In the centuries preceding European Renaissance knowledge in medicine and many other areas tended to be rather syncretic. It represented a body of knowledge integrated into the existing religious system and a perception of life. Probably the most representative example is the Persian philosopher Avicenna’s encyclopedia “The Canon of Medicine.” However, since the awakening of scientific way of thinking in the fifteenth century Europe, there has been a huge blossoming of knowledge that tended to narrowly specialize.

These long-term changes in build-up and practical application of scientific knowledge underwent a huge extent of overspecialization. In such a gnostic evolution, it became obvious that certain, originally related disciplines, moved so much away from each other. They lost both mutual understanding and complementarity in real-life applications (1).

The mainstream of scientific development already had thousands of branch disciplines as we approached the twenty-first century. It became obvious that this pose a serious obstacle to further meaningful development (2). Modern day thinkers and researchers are finding it harder than ever to grasp the big picture in their areas of endeavor (3). A need for building bridges among the existing sciences emerged. In fact, it was very early embraced as the concept of interdisciplinarity (4).

With this bibliographic piece our effort was aimed at observing the quantitative scale of evidence on publishing output in one exemplary mature interdisciplinary science. We decided to observe health economics for several reasons. The first reason is that the need for interdisciplinary research was early recognized in health sciences in decades following the World War II (5). The second reason is the fact that integration between medicine and social sciences recorded bold growth during the twentieth century (6, 7). And last, but not the least, health economics itself presents a convenient example as probably one of the most developed sciences bridging this gap from a historical perspective (8).

## Methods

The methods we relied on were chosen to show rather simple crosscuts of academic publishing in the area, while adopting time horizon from January 1, 2000 to December 31, 2016. We focused on comparing quantitative outputs in health economics across four different databases. Electronic search of the published literature was conducted in one medical database—MEDLINE/PubMed, two general databases—Scopus/Elsevier and Web of Science (WoS), and one specialized health economic database—NHS Economic Evaluation Database (EED). NHS EED contains economic evaluations of health-care interventions (cost–benefit analyses, cost–utility analyses, and cost-effectiveness analyses) and was produced by the NIHR Centre for Reviews and Dissemination at the University of York, United Kingdom (9). Funding for producing NHS EED ceased at the end of March 2015, whereas electronic searches for compiling the database were continued until the end of the 2014.

### Search Strategy

The search strategies for each database are presented in detail in the Data Sheet S1 in Supplementary Material. Electronic searches were conducted until July 15, 2017. The applied combination of key words was carefully chosen to cover the most commonly used terms in the titles of publications dealing with conceptual areas of health economics. We tried to ensure inclusion of the largest possible number of the publications really dealing with health economics, and on the other hand exclusion of the largest possible number of irrelevant publications. Each time we considered to include or exclude a key word we reviewed the first 100 identified records in order to evaluate whether their main topic belongs to the health economics area. Final applied combination included 88 key words combined with the Boolean search operators “OR” and “AND.” This combination of key words was used across three databases (MEDLINE/PubMed, Scopus, and WoS). Appropriate operator, as instructed in each database, was used to limit finding key words only in the titles of the records. There were no restrictions regarding countries where authors’ affiliations are based or language of full text publishing. All bibliographic units were taken into account (articles, reviews, books, dissertations, etc.). No filter was applied in the MEDLINE database. In the general databases (Scopus/Elsevier and WoS) filters related to medical and economics subject areas were applied as indicated in the search strategy in the Data Sheet S1 in Supplementary Material. Since NHS EED database contains only economic evaluations of health economic interventions, only publication year filter was applied and key words were not used in the search. Also, we used feature provided by Scopus/Elsevier and WoS databases to additionally analyze identified records by the fields Country/Territory and Organizations (in WoS) and Affiliations (in Scopus) of authors.

## Results

**Figure 1:**
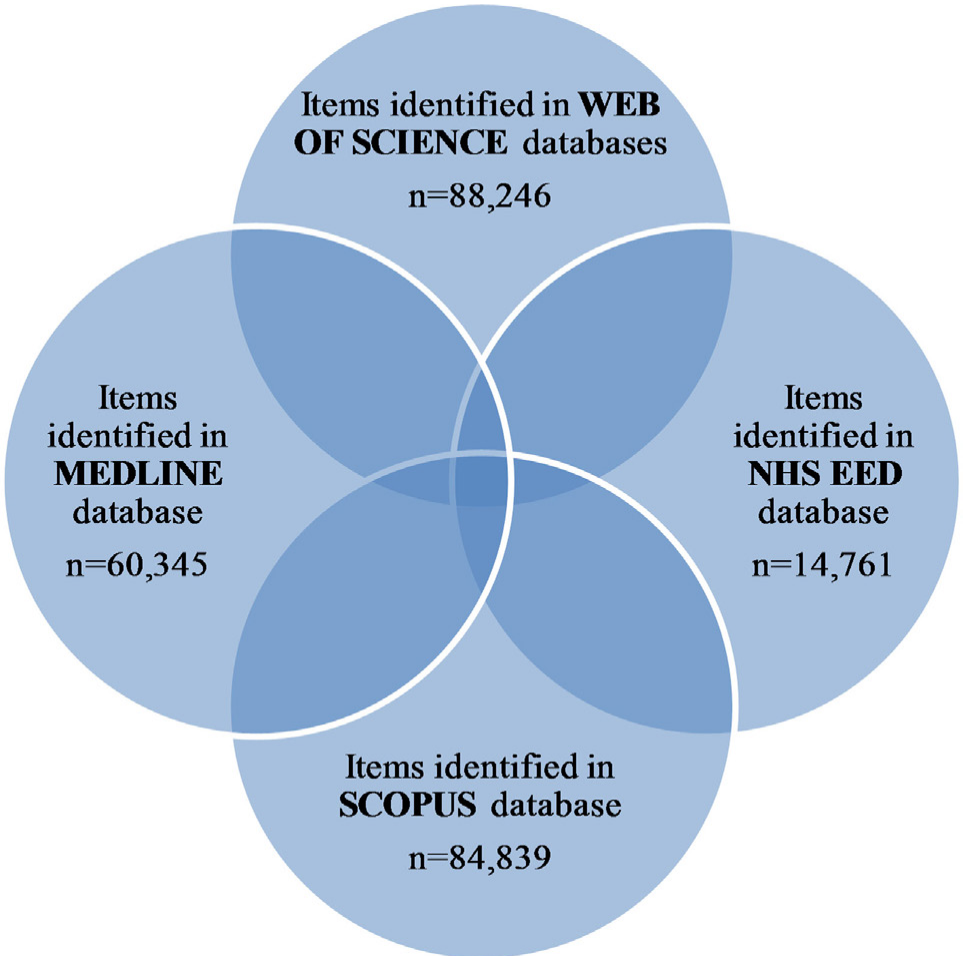
Selection of publications.

Results of the literature search are shown in the Table 1 and Figure 1. Numbers which are presented there depict number of identified records in the given year with applied search strategy in each database. The largest absolute number of records for 2000–2016 time span was detected in the WoS database (88,246). The smallest number of records (14,761) was detected in the NHS EED, and this number reflects only economic evaluations of health interventions (cost–benefit analyses, cost–utility analyses, and cost-effectiveness analyses), which satisfied criteria for inclusion in this database. As the electronic searches for production of NHS EED were conducted until the end of the 2014 and the database was no longer updated after the funding was stopped, number of records in 2014 and afterward may not reflect actual output of the health economic evaluations for that period. Annual number of records was similar across WoS and Scopus, whereas slightly smaller annual number of records was observed in MEDLINE. We can observe that number of records increased over the years.

**Table 1:** Approximate quantitative output in diverse health economics areas worldwide is presented across four major indexing databases in chronological order below.

Year	Web of Science	Scopus	MEDLINE	NHS Economic Evaluation Database
2000	2,577	2,794	2,145	600
2001	2,371	2,805	2,169	585
2002	2,797	2,875	2,281	622
2003	3,240	3,356	2,501	671
2004	3,432	3,338	2,577	640
2005	3,747	3,577	2,645	725
2006	3,940	3,937	2,790	800
2007	4,419	4,291	2,773	867
2008	4,915	4,648	3,034	1,033
2009	5,471	5,058	3,250	1,023
2010	5,520	5,504	3,532	1,075
2011	6,047	5,800	3,689	1,224
2012	6,819	6,535	4,270	1,551
2013	7,411	7,081	4,671	1,956
2014	7,843	7,652	5,913	1,386
2015	8,704	7,867	6,412	3
2016	8,993	7,721	5,693	0

Analysis of the identified records by Country/Territory field in WoS and Scopus is presented in the Table 2. With slightly more than one-third of the identified records, USA clearly dominates in this field. United Kingdom takes a strong second place with about 12% of identified records. Majority of other countries in the top 15 are high-income European countries (Germany, the Netherlands, France, Spain, Italy, Switzerland, Sweden, and Belgium), along with Canada, Australia, China, Brazil, Japan, and India with contribution which varies from 1.5 to 6.2% of identified records. If we expand our analysis to the top 50 countries/territories, we can observe that about one-third of them belong to the middle-income group according to the World Bank list of economies, while remaining share belongs to the high-income group (Data Sheet S2 in Supplementary Material). Consistently, USA and UK universities are the most frequent among the top 15 affiliations/organizations of the authors of the identified records with slight variations in the rank order between the two databases (Table 3). Among the top 15 are also one Canadian university (University of Toronto) and one Dutch university (Erasmus University Rotterdam), as well as one multinational pharmaceutical company (Pfizer Inc.). Authors from Harvard University contributed to the largest number of the identified records. List of the top 50 Affiliations/Organization of the authors of the identified records is provided in the Data Sheet S2 in Supplementary Material.

**Table 2:** Representation of defined countries/territories associated with identified records in Web of Science (WoS) and Scopus (the top 15 countries/territories shown).

	WoS		Scopus	
	Country/territory	Number of records (%)	Country/territory	Number of records (%)
1	USA	34,779 (39.4)	USA	29,116 (34.3)
2	England	10,581 (12.0)	United Kingdom	9,853 (11.6)
3	Canada	5,106 (5.8)	Germany	5,296 (6.2)
4	Germany	4,955 (5.6)	Canada	4,344 (5.1)
5	The Netherlands	3,808 (4.3)	Australia	3,324 (3.9)
6	France	3,410 (3.9)	The Netherlands	3,285 (3.9)
7	Australia	3,343 (3.8)	France	3,051 (3.6)
8	Spain	3,006 (3.4)	Spain	2,853 (3.4)
9	Italy	2,924 (3.3)	Italy	2,707 (3.2)
10	China	2,856 (3.2)	Switzerland	1,977 (2.3)
11	Switzerland	2,545 (2.9)	China	1,967 (2.3)
12	Sweden	1,999 (2.3)	Sweden	1,698 (2.0)
13	Belgium	1,812 (2.1)	India	1,544 (1.8)
14	Brazil	1,620 (1.8)	Belgium	1,407 (1.7)
15	Japan	1,295 (1.5)	Japan	1,380 (1.6)

**Table 3:** Representation of affiliations/organizations of the authors of the identified records in Web of Science (WoS) and Scopus (the top 15 affiliations/organizations shown).

	WoS		Scopus	
	Organization	Number of records (%)	Affiliation	Number of records (%)
1	Harvard University	2,125 (2.4)	Harvard Medical School	1,124 (1.3)
2	University of Toronto	1,044 (1.2)	VA Medical Center	1,077 (1.3)
3	University of Washington	1,016 (1.2)	University of Toronto	945 (1.1)
4	University of Michigan	990 (1.1)	London School of Hygiene & Tropical Medicine	787 (0.9)
5	University of California, San Francisco	903 (1.0)	Centers for Disease Control and Prevention	768 (0.9)
6	University of York	788 (0.9)	Harvard School of Public Health	765 (0.9)
7	University of Pennsylvania	717 (0.8)	University of Washington, Seattle	759 (0.9)
8	University of California, Los Angeles	701 (0.8)	University of California, San Francisco	748 (0.9)
9	Stanford University	697 (0.8)	University of York	739 (0.9)
10	Johns Hopkins University	685 (0.8)	University of Oxford	627 (0.7)
11	Duke University	681 (0.8)	Pfizer Inc.	613 (0.7)
12	Erasmus University Rotterdam	673 (0.8)	Brigham and Women’s Hospital	601 (0.7)
13	University of North Carolina	650 (0.7)	University of Pennsylvania	571 (0.7)
14	Centers for Disease Control and Prevention	649 (0.7)	Erasmus University Medical Center	558 (0.7)
15	University of Oxford	613 (0.7)	King’s College London	554 (0.7)

## Discussion

There were several serious attempts to grasp a development of academic publishing in health economics. We would like to point out two prominent examples: Rubin and Chang in 2003 (10) and Wagstaff and Culyer in 2012 (11). Both were extraordinary bibliographic research efforts. The first one concentrated on EconLit using JEL codes on time horizon 1991–2000 processing ~5,500 articles. The latter had far broader time horizon (1969–2009) and processed ~33,000 articles by relying on EconLit and JEL codes as well. However, analysis based only on EconLit database has an important limitation (10). EconLit encompasses a wide range of economics and business journals, but it does not index numerous social welfare, health-care and biomedical journals that publish a significant number of health economics articles (10). This disparity was particularly noticed in a recent bibliometric analysis of economic evaluations of health interventions by Pitt et al. (12). This analysis identified 2,844 full economic evaluations which met predefined set of criteria by searching 14 databases for articles published between January 2012 and May 2014 (12). EconLit database captured only 1% of all identified economic evaluations in this analysis (12).

Our search strategy identified an increasing number of health economics related records across the four databases. However, it should be noted that the numbers presented in the results provide only an estimate of the growth of the health economics publications, as more detailed analysis of all identified records was precluded. Excluding NHS EED, search of remaining three databases relying on the combination of key words and categories (where category filter was available) carries a risk of omitting genuine health economic publications as well as including those that perhaps are not related to the field. Similar limitation was noted when relying on health JEL codes in EconLit as was the case in previous bibliographic efforts (11). The authors acknowledged that health economics publications could be missed when the author did not choose health JEL code even if the publication contains substantial amount of material on health, or irrelevant publications could be included if the article, despite having a health JEL code, contains small or negligible content on health (11).

USA was identified as the top country in health economics research, followed by the UK. Of all identified institutions, Harvard University seems to be a leader in this field. This finding is consistent with previous reviews despite differences in methodology (11, 12). However, middle-income countries are also becoming more noticeable. As pointed out by some earlier investigators, it is evident that health economics productivity is shifting its geographic outreach from mostly Western, OECD economies, toward the low and middle-income countries worldwide (13). This profound change is aligned with the global shift of health-care spending in the same direction, particularly since the beginning of the twenty-first century (14). Changes in priority of the governmental health-care investment and research funding for health economics are most visible when comparing the top emerging BRICS with G7 nations (15, 16).

In addition, this short bibliographic insight reveals one key issue. The conditions aimed at supporting social drivers of research which connects medicine and social sciences are successfully leading to the long-run outcomes. Societal imperative to increase cost-effective resource allocation in health care becomes more obvious. Heavy burden of population aging and prosperity diseases posed on contemporary societies is certainly a substantial contributing factor. Even the richest of OECD nations are facing the challenge of financial sustainability regarding health share of national GDP.

The broad area of interdisciplinary research continues to develop. In response to this, supranational authorities recognized the need to invest in its fostering. Prime example of such funding priorities is a grant funded by the European Commission—INTREPID COST action which is a network of 27 countries established with the aim to better understand how to achieve more efficient and effective interdisciplinary research in Europe (17). Similar initiatives have spread across the globe and include noticeable grants of the US federal agencies (18–21) and Japan (22). In this sense, our example with health economics should only depict the same mainstream process of bridging scientific knowledge that happens simultaneously elsewhere on a number of crossroads among diverse disciplines (23). However, there are also the opposed concerning tendencies affecting social interdisciplinary scientists who claim to be underfunded or that such proposals are significantly less likely to get funded (24). These and similar trends should raise attention of policy makers against such rooted practice in many funding agencies (25). Broad societal perspective on gains and losses from narrow and deep overspecialization of research could only be provided by strong interdisciplinary development (26).

## Conclusion

We may conclude that there is a clear evidence of rise in global quantitative output of academic publishing in interdisciplinary science of health economics. Each of the large databases grasps another angle of this research proliferation. MEDLINE is leaning toward applications in clinical medicine. Scopus and WoS are somewhere in between, catching slightly different cross-sections of both economics and medicine. NHS EED is probably the most precisely matching academic research growth in health economics, although with the risk of omitting borderline materials published elsewhere, outside of reach of this registry. However, in all four registries we have evidence of bold rise in research output. This example might serve as a promising one for further interdisciplinary development in other areas (27).

## Author Contributions

MJ and AP have jointly designed the research question, prepared the manuscript, and revised it for important intellectual content.

## Conflict of Interest Statement

The authors declare that the research was conducted in the absence of any commercial or financial relationships that could be construed as a potential conflict of interest.
