# Supplementary material for: Growth of Global Publishing Output of Health Economics in the Twenty-First Century: A Bibliographic Insight
Source: Front Public Health. 2017 Aug 11;5:211. doi: 10.3389/fpubh.2017.00211 (PMC5554506; doi:10.3389/fpubh.2017.00211)
Supplement: Supplementary file 1 [file data_sheet_1.docx]

## Supplementary file 1

## Search strategy

### MEDLINE/Pubmed

(economic[ti] OR economics[ti] OR economy[ti] OR economical*[ti] OR pharmacoeconomic*[ti] OR cost[ti] OR costs[ti] OR expenditure[ti] OR expenditures[ti] OR budget[ti] OR expense[ti] OR expenses[ti] OR market[ti] OR spend[ti] OR spends[ti] OR spending[ti] OR payment[ti] OR payments[ti] OR out-of-pocket[ti]) AND (burden[ti] OR disease[ti] OR diseases[ti] OR care[ti] OR driver[ti] OR drivers[ti] OR treatment[ti] OR treatments[ti] OR treating[ti] OR treated[ti] OR treat[ti] OR management[ti] OR pattern[ti] OR patterns[ti] OR inpatient[ti] OR outpatient[ti] OR hospital[ti] OR medical[ti] OR insurance[ti] OR out-of-pocket[ti] OR service[ti] OR services[ti] OR drug[ti] OR drugs[ti] OR pharmaceutical[ti] OR pharmaceuticals[ti] OR resource[ti] OR resources[ti] OR therapy[ti] OR coverage[ti] OR health[ti] OR healthcare[ti] OR health-care[ti] OR diagnosis[ti] OR diagnostic[ti] OR medicine[ti] OR medicines[ti] OR medication[ti] OR prevention[ti] OR vaccination[ti] OR vaccine[ti] OR screening[ti] OR screen[ti] OR analysis[ti] OR vaccine[ti] OR utilization[ti] OR allocation[ti] OR illness[ti] OR illnesses[ti] OR evaluation[ti] OR impact[ti] OR saving[ti] OR savings[ti] OR containment[ti] OR prophylaxis[ti] OR surgery[ti] OR rehabilitation[ti] OR generic[ti] OR generics[ti] OR biosimilar[ti] OR biosimilars[ti] OR injection[ti] OR injections[ti] OR implant[ti] OR implants[ti] OR implantation[ti] OR psychiatry[ti] OR technology[ti] OR assessment[ti])

### SCOPUS

TITLE ( economic OR economics OR economy OR economical* OR pharmacoeconomic* OR cost OR costs OR expenditure OR expenditures OR budget OR expense OR expenses OR market OR spend OR spends OR spending OR payment OR payments OR out-of-pocket ) AND TITLE ( burden OR disease OR diseases OR care OR driver OR drivers OR treatment OR treatments OR treating OR treated OR treat OR management OR pattern OR patterns OR inpatient OR outpatient OR hospital OR medical OR insurance OR out-of-pocket OR service OR services OR drug OR drugs OR pharmaceutical OR pharmaceuticals OR resource OR resources OR therapy OR coverage OR health OR healthcare OR health-care OR diagnosis OR diagnostic OR medicine OR medicines OR medication OR prevention OR vaccination OR vaccine OR screening OR screen OR analysis OR vaccine OR utilization OR allocation OR illness OR illnesses OR evaluation OR impact OR saving OR savings OR containment OR prophylaxis OR surgery OR rehabilitation OR generic OR generics OR biosimilar OR biosimilars OR injection OR injections OR implant OR implants OR implantation OR psychiatry OR technology OR assessment ) AND ( LIMIT-TO ( PUBYEAR , 2016 ) OR LIMIT-TO ( PUBYEAR , 2015 ) OR LIMIT-TO ( PUBYEAR , 2014 ) OR LIMIT-TO ( PUBYEAR , 2013 ) OR LIMIT-TO ( PUBYEAR , 2012 ) OR LIMIT-TO ( PUBYEAR , 2011 ) OR LIMIT-TO ( PUBYEAR , 2010 ) OR LIMIT-TO ( PUBYEAR , 2009 ) OR LIMIT-TO ( PUBYEAR , 2008 ) OR LIMIT-TO ( PUBYEAR , 2007 ) OR LIMIT-TO ( PUBYEAR , 2006 ) OR LIMIT-TO ( PUBYEAR , 2005 ) OR LIMIT-TO ( PUBYEAR , 2004 ) OR LIMIT-TO ( PUBYEAR , 2003 ) OR LIMIT-TO ( PUBYEAR , 2002 ) OR LIMIT-TO ( PUBYEAR , 2001 ) OR LIMIT-TO ( PUBYEAR , 2000 ) ) AND ( LIMIT-TO ( SUBJAREA , "MEDI" ) OR LIMIT-TO ( SUBJAREA , "ECON" ) OR LIMIT-TO ( SUBJAREA , "PHAR" ) OR LIMIT-TO ( SUBJAREA , "BIOC" ) OR LIMIT-TO ( SUBJAREA , "NURS" ) OR LIMIT-TO ( SUBJAREA , "HEAL" ) OR LIMIT-TO ( SUBJAREA , "IMMU" ) OR LIMIT-TO ( SUBJAREA , "NEUR" ) OR LIMIT-TO ( SUBJAREA , "DENT" ) )

### WEB OF SCIENCE

TI=(economic OR economics OR economy OR economical* OR pharmacoeconomic* OR cost OR costs OR expenditure OR expenditures OR budget OR expense OR expenses OR market OR spend OR spends OR spending OR payment OR payments OR out-of-pocket) AND TI=(burden OR disease OR diseases OR care OR driver OR drivers OR treatment OR treatments OR treating OR treated OR treat OR management OR pattern OR patterns OR inpatient OR outpatient OR hospital OR medical OR insurance OR out-of-pocket OR service OR services OR drug OR drugs OR pharmaceutical OR pharmaceuticals OR resource OR resources OR therapy OR coverage OR health OR healthcare OR health-care OR diagnosis OR diagnostic OR medicine OR medicines OR medication OR prevention OR vaccination OR vaccine OR screening OR screen OR analysis OR vaccine OR utilization OR allocation OR illness OR illnesses OR evaluation OR impact OR saving OR savings OR containment OR prophylaxis OR surgery OR rehabilitation OR generic OR generics OR biosimilar OR biosimilars OR injection OR injections OR implant OR implants OR implantation OR psychiatry OR technology OR assessment)

**Refined by:** **WEB OF SCIENCE CATEGORIES:** ( ECONOMICS OR UROLOGY NEPHROLOGY OR HEALTH CARE SCIENCES SERVICES OR HEMATOLOGY OR HEALTH POLICY SERVICES OR OBSTETRICS GYNECOLOGY OR PUBLIC ENVIRONMENTAL OCCUPATIONAL HEALTH OR MEDICINE GENERAL INTERNAL OR RESPIRATORY SYSTEM OR PERIPHERAL VASCULAR DISEASE OR PHARMACOLOGY PHARMACY OR PEDIATRICS OR TRANSPLANTATION OR ONCOLOGY OR RHEUMATOLOGY OR SURGERY OR GERIATRICS GERONTOLOGY OR SOCIAL SCIENCES BIOMEDICAL OR DERMATOLOGY OR BIOTECHNOLOGY APPLIED MICROBIOLOGY OR NURSING OR CARDIAC CARDIOVASCULAR SYSTEMS OR CRITICAL CARE MEDICINE OR REHABILITATION OR CLINICAL NEUROLOGY OR GASTROENTEROLOGY HEPATOLOGY OR RADIOLOGY NUCLEAR MEDICINE MEDICAL IMAGING OR PSYCHIATRY OR INFECTIOUS DISEASES OR NEUROSCIENCES OR TROPICAL MEDICINE OR IMMUNOLOGY OR MICROBIOLOGY OR ENDOCRINOLOGY METABOLISM OR GERONTOLOGY OR ORTHOPEDICS OR SUBSTANCE ABUSE OR MEDICAL INFORMATICS OR OPHTHALMOLOGY OR NUTRITION DIETETICS OR MEDICINE RESEARCH EXPERIMENTAL )

**Timespan:** 2000-2016. **Indexes:** SCI-EXPANDED, SSCI, A&HCI, CPCI-S, CPCI-SSH, ESCI.

### NHS ECONOMIC EVALUATION DATABASE (EED)

(* ) and ((Economic evaluation:ZDT and Bibliographic:ZPS) OR (Economic evaluation:ZDT and Abstract:ZPS)) IN NHSEED FROM 2000 TO 2016
